# Induction of Cancer Cell Death by Isoflavone: The Role of Multiple Signaling Pathways

**DOI:** 10.3390/nu3100877

**Published:** 2011-10-17

**Authors:** Yiwei Li, Dejuan Kong, Bin Bao, Aamir Ahmad, Fazlul H. Sarkar

**Affiliations:** Department of Pathology, Barbara Ann Karmanos Cancer Institute, Wayne State University School of Medicine, 740 Hudson Webber Cancer Research Center, 4100 John R, Detroit, MI 48201, USA; Email: yiweili@med.wayne.edu (Y.L.); dkong@med.wayne.edu (D.K.); baob@karmanos.org (B.B.); ahmada@karmanos.org (A.A.)

**Keywords:** cell signaling, apoptosis, isoflavone

## Abstract

Soy isoflavones have been documented as dietary nutrients broadly classified as “natural agents” which plays important roles in reducing the incidence of hormone-related cancers in Asian countries, and have shown inhibitory effects on cancer development and progression *in vitro* and *in vivo*, suggesting the cancer preventive or therapeutic activity of soy isoflavones against cancers. Emerging experimental evidence shows that isoflavones could induce cancer cell death by regulating multiple cellular signaling pathways including Akt, NF-κB, MAPK, Wnt, androgen receptor (AR), p53 and Notch signaling, all of which have been found to be deregulated in cancer cells. Therefore, homeostatic regulation of these important cellular signaling pathways by isoflavones could be useful for the activation of cell death signaling, which could result in the induction of apoptosis of both pre-cancerous and/or cancerous cells without affecting normal cells. In this article, we have attempted to summarize the current state-of-our-knowledge regarding the induction of cancer cell death pathways by isoflavones, which is believed to be mediated through the regulation of multiple cellular signaling pathways. The knowledge gained from this article will provide a comprehensive view on the molecular mechanism(s) by which soy isoflavones may exert their effects on the prevention of tumor progression and/or treatment of human malignancies, which would also aid in stimulating further in-depth mechanistic research and foster the initiation of novel clinical trials.

## 1. Introduction

Cancer cells are known to exhibit deregulations in multiple signaling pathways, leading to uncontrolled cell proliferation and acquired anti-apoptosis features. Apoptosis also known as programmed cell death, an important physiological process, which occurs in cells during development and normal cellular processes. It contains sequential responses to biochemical or physical signals to maintain cellular homeostasis because the damaged and useless cells must die through apoptotic process for their elimination. It has been known that cells altered beyond repair by normal mechanisms or cells that have completed their biological function proceed to the apoptosis process [1]. Apoptosis is induced by several cellular signals which alter mitochondrial permeability, leading to a cascade of events such as the release of apoptosis activators from mitochondria, which leads to the activation of caspases (cysteinyl-aspartate-specific proteinases), a family of intracellular cysteine proteases causing apoptotic cell death [2,3,4]. The activation of caspases then targets their downstream molecules [2,3,4]. Poly-ADP-Ribose-Polymerase (PARP) is one of the targets of caspases and specifically binds at DNA strand breaks. The activated caspases cleave PARP to two fragments during early stages of apoptosis; therefore, the cleavage of PARP is an early marker of apoptosis [5,6]. Other important genes involved in the apoptotic processes include Bcl-2, Bcl-XL, Bax, p53, *etc*. [7,8,9]. Among them, Bcl-2 and Bax are known to play critical roles in apoptosis, and most interestingly the ratio of Bcl-2 and Bax reflects the status of apoptosis.

During the apoptotic process, another important cellular response is the activation of endogenous Ca^2+^ and Mg^2+^-dependent nuclear endonuclease [10]. In human chromosome, histone and DNA strand tightly bind to form nucleosomal units, and each unit includes ~180-200 bp DNA and histone protein. When apoptosis occurs, the activated endonuclease selectively cleaves DNA at sites located between nucleosomal units, generating typical ~180-200 bp × *n* DNA fragments with histone and eventually leading to changes in the morphological features [11]. These features include chromatin aggregation, nuclear and cytoplasmic condensation, partition of cytoplasm and nucleus into membrane bound-vesicles that are known as apoptotic bodies. *In vivo*, these apoptotic bodies are rapidly recognized and phagocytosed by either macrophages or adjacent epithelial cells. In this way, the unwanted or damaged cells are eliminated to maintain the normal physiological homeostasis. However, if the cellular signals for initiating apoptosis are lost, a variety of malignant disorders may result. Development and differentiation of normal tissue are controlled by a balance between cell proliferation and apoptosis, and defects in such balance leads to the development of cancers because of uncontrolled cell proliferation and reduced apoptotic cell death [12,13]. In cancers, un-repaired damaged cells survive and grow, resulting in uncontrolled cancer growth, invasion and metastasis. It is now generally accepted that defective apoptosis mechanism plays important roles in carcinogenesis and cancer progression. 

It is important to note that the activators and inhibitors of apoptosis are also the molecules distributed in various cellular signaling pathways. Cellular signaling is a complex signal communication network which controls biological activities of cells and coordinates cellular function including apoptosis. The signaling cascades are typically composed of three-dimensional pathways of proteins which regulate each other within a specific sub-cellular location in the cells [14]. Because of the complex transduction of cell signal, cancer cells always exhibit the alterations in multiple cellular signaling pathways. It is known that several important cellular signaling pathways including NF-κB, Akt, MAPK, Wnt, Notch, p53, AR, *etc*., control apoptosis and cell proliferation. Importantly, all of these signaling pathways have been found malfunctioning in cancer cells, resulting in defective apoptosis, increased tumor growth, invasion, and metastasis [15,16,17,18,19]. Moreover, the ability of cancer cells to evade apoptosis also plays a critical role in *de novo* (intrinsic) and acquired (extrinsic) resistance to conventional therapeutics [13,20]. Therefore, it is important to design a strategy that could simultaneously target multiple cellular signaling pathways, which will lead to increased apoptotic cell death and enhanced cell growth inhibition in order to eliminate cancer cells.

Recently, dietary compounds have received much attention in the field of cancer prevention and therapy, primarily because epidemiological studies have shown that the consumption of fruits, soybean and vegetables is associated with reduced risk of several types of cancers [21,22,23]. The dietary compounds including isoflavone-genistein, indole-3-carbinol (I3C), 3,3′-diindolylmethane (DIM), curcumin, (−)-epigallocatechin-3-gallate (EGCG), resveratrol, lycopene, *etc*., have been recognized as cancer chemopreventive agents because of their anti-carcinogenic activity [24,25]. Moreover, the *in vitro *and *in vivo *studies have demonstrated that these dietary compounds have inhibitory effects on various human and animal cancers [26,27,28,29]. Furthermore, experimental studies have demonstrated that these dietary compounds can enhance the anti-tumor activity of chemotherapeutic agents by regulation of cellular signal transduction pathways, resulting in the induction of apoptotic cell death. Among these compounds, isoflavones show their strong effects on apoptosis pathways. Soy isoflavones such as genistein, daidzein, and glycitein are mainly derived from soybean. Isoflavone-genistein has been found to induce apoptotic cell death *in vivo* and *in vitro* [30,31]. Emerging evidence from increasing number of investigations on isoflavones has shown that isoflavones exert their pleiotropic effects on cancer cells through targeting multiple cellular signaling pathways including NF-κB, Akt, MAPK, Wnt, Notch, p53, and AR pathways, suggesting that isoflavone could be useful either alone or in combination with conventional therapeutics for the prevention of tumor progression and/or treatment of human malignancies.

## 2. Deregulation of Cellular Signaling and Apoptosis Pathway in Cancer Cells

In cancer cells, altered proteins produced due to mutations, other defects, or amplifications of genes, impact cellular signal communication which controls apoptotic cell death. NF-κB, Akt, MAPK, Wnt, Notch, p53, and AR pathways are commonly deregulated in various cancers as discussed in-depth in the following sections.

**Figure 1:**
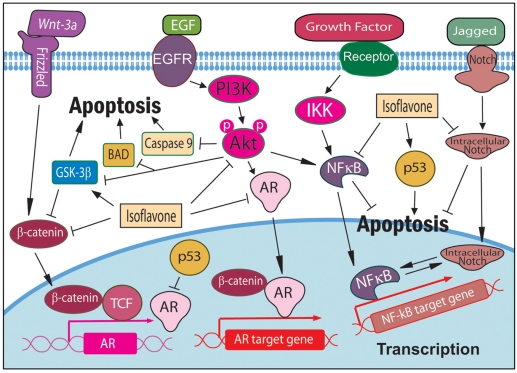
Cellular signaling pathways involved in the induction of apoptosis by isoflavone.

Nuclear factor-κB (NF-κB) signaling pathway plays important roles in the control of cell growth, apoptosis, inflammation, stress response, and many other physiological processes [16,32,33]. Several important molecules such as NF-κB, IκB, and IKK in the NF-κB signaling pathway regulate apoptotic signal transduction; however, NF-κB is the key protein in the pathway and has been described as a major culprit and a therapeutic target in cancer [34,35]. The activation of NF-κB is frequently observed in various cancer cells. The constitutive activation of NF-κB observed in cancer cells is likely due to the involvement of multiple other signal transduction pathways such as tyrosine kinase, NIK, and Akt pathways. It is known that NF-κB is a key modulator of apoptosis in a variety of cell types. Activation of NF-κB inhibits apoptosis while inhibition of NF-κB sensitizes human cancer cells to apoptosis [36,37], suggesting that NF-κB signaling plays important roles in apoptotic pathway (Figure 1).

Experimental studies have demonstrated the crosstalk between NF-κB and Akt signaling [38,39]. Akt signaling pathway also plays critical roles in cell survival regulation and is frequently activated in various cancers [40,41]. Akt is activated by cellular survival signals, leading to phosphorylation at Thr308 and Ser473 [42]. Activated Akt functions to promote cell survival by inhibiting apoptosis through inactivation of several pro-apoptotic factors including Bad, Forkhead transcription factors, and caspase-9 (Figure 1) [43,44,45]. Studies have also shown that Akt regulates the NF-κB pathway via phosphorylation and activation of molecules in the NF-κB signaling pathway [46,47], leading to the inhibition of apoptosis. Therefore, Akt is another attractive target for cancer prevention or treatment [48] because inactivation of Akt signaling could revert anti-apoptotic status in cancer cells via inhibition of pro-apoptotic factor and NF-κB.

MAPK signaling is another signaling pathway which has received increasing attention as a target for cancer prevention and treatment. MAPK signaling includes a three-tiered kinase core where MAP3K activates MAP2K that activates MAPKs (ERK, JNK, and p38), leading to the activation of NF-κB, cell growth, and cell survival [49,50]. It has been reported that MAPK is activated in various cancer and that the activation of MAPK is also linked to cancer progression including angiogenesis, invasion, and metastasis [51]. The reported roles of MAPK signaling in apoptotic cell death are controversial. It has been reported that blocking the MEK/ERK signaling using the small-molecule MAPK inhibitors significantly enhances arsenic trioxide (ATO)-induced apoptosis in human myeloma cell lines [52]. Further studies showed that the inhibition of both MAPK and NF-κB signaling is necessary for rapid apoptosis in macrophages [53]. However, other reports also showed that activation of p38 MAPK was required for Bax translocation to mitochondria, cytochrome c release and apoptosis induced by UVB irradiation in human keratinocytes [54] and that protein kinase C induced apoptosis in LNCaP prostate cancer cells was mediated through the activation of p38 MAPK and inhibition of Akt pathway [55]. Therefore, further investigations are needed to resolve some existing controversies on the role of MAPK signaling in apoptosis pathway.

Recent evidence has demonstrated that Wnt signaling plays important roles in cancer development and progression. Wnt signaling critically participates in the embryonic developmental processes including cell proliferation, differentiation, apoptosis and epithelial-mesenchymal interactions [56]. The aberrant activation of the canonical Wnt/β-catenin signaling is one of the most frequent signaling abnormalities known in human malignancy. In human cancers, activated Wnt signal promotes β-catenin accumulation in the nucleus, leading to the transcriptional activation of specific target genes which promote cancer progression (Figure 1) [57,58]. The aberrant expression of Wnt ligand and Wnt binding proteins and the activation of the Wnt signaling have been found in a variety of human tumors [59]. Experimental studies also showed that activation of Wnt signaling inhibited apoptosis in cancer cells. It has been found that Wnt signaling inhibits FOXO3a-induced transcription and apoptosis in colon cancer [60] and that Wnt proteins prevents apoptosis of osteoblasts through β-catenin, Src/ERK, and Akt signaling pathways [61]. Moreover, studies have shown that inactivation of Wnt signaling could induce apoptosis in colon cancer and melanoma [62,63]. Therefore, inhibition of aberrant Wnt activity in cancer cells could provide an opportunity for cancer treatment [64,65].

Similar to Wnt signaling, Notch signaling also participates in the embryonic developmental processes. Proper functioning of Notch signaling is required for normal development during early life. It has been found that Notch signaling plays critical roles in the regulation and maintenance of stem cells. Importantly, growing evidence demonstrates that deregulation of Notch signaling could lead to the development and progression of various cancers [66,67]. Up-regulation of Notch receptors and their ligands has been observed in lymphomas, cervical, lung, colon, head and neck, renal, and pancreatic cancers [66,68,69]. In cancer cells, Notch signaling is abnormally activated, leading to decreased apoptosis, increased proliferation of cancer cells, and induced Epithelial-to-Mesenchymal Transition (EMT), a process that is reminiscent of cancer stem-like cell characteristics (Figure 1). We and other investigators have found that Notch activation could induce pro-apoptosis molecules by activation of Akt or NF-κB signaling or by causing interference with XIAP ubiquitination and degradation [69,70,71,72]. Thus, the inhibition of Notch signaling could induce apoptosis and could also enhance sensitivity of cancer cells to conventional chemotherapeutics [73,74]. Therefore, Notch signaling pathway is now believed to be an important target for cancer therapy. However, few reports showed that Notch signaling could be a potent inducer of growth arrest and apoptosis [75,76], suggesting that the Notch signaling could be context-dependent, and thus further in-depth investigations are needed to address the existing controversies on the role of Notch as a target for cancer therapy.

The p53 gene is a well-known transcription factor and tumor suppressor, which critically participates in many cellular processes including apoptosis, cell signal transduction, cellular response to DNA-damage, genomic stability, and cell cycle control (Figure 1). Wild-type p53 functions as a tumor suppressor and activates the transcription of its downstream genes such as p21^WAF1^ and Bax to induce apoptosis and inhibit proliferation of DNA damaged cells or cancer cells. Importantly, the mutations and the inactivation of p53 have been found in various cancers and the status of p53 in cancer cells determines the cellular response to chemotherapy [77], suggesting that normalization of wild-type p53 function could be a novel approach for the treatment of cancer. 

Androgen receptor (AR) is a ligand-activated transcription factor of the nuclear receptor superfamily that plays a critical role in male physiology and pathology, and also beginning to be realized for many functions in female as well. It has been well accepted that AR signaling contributes to carcinogenesis and cancer progression through regulating transcription of androgen-responsive genes (Figure 1) [78]. The deregulated AR signaling is commonly observed in cancer cells, especially prostate cancer cells. It is known that androgen and AR are involved in all stages of prostate cancers including initiation, progression, and treatment resistance [79], suggesting that AR signaling contributes to anti-apoptotic mechanism. Moreover, one of the androgen-responsive genes, prostate specific antigen (PSA), is a clinically important marker that is routinely used for diagnosis, monitor treatment response, prognosis and disease progression in patients diagnosed with prostate cancer [80,81]. Furthermore, during the progression of prostate cancer from androgen sensitive status to castrate resistant prostate cancer, the majority of prostate cancer cells still expresses AR, suggesting that AR signaling plays a critical role in the development and progression of prostate cancer [82]. Therefore, AR signaling has long been recognized as a main target for prostate cancer prevention and treatment. AR could also exhibit pro- or anti-apoptosis activity in different cellular situation. Androgen and AR could promote Bax-mediated apoptosis [83]; however, down-regulation of AR by synthetic or natural agents could also induce apoptosis and inhibit cancer cell proliferation [84,85]. These results clearly suggest that the AR signaling pathway is a complex signaling network, and thus further in-depth research in this area is an active area of investigation not only for prostate cancer but also for breast and other cancers.

## 3. Regulation of Cellular Signaling to Induce Apoptosis by Isoflavone

The cellular signaling is a complex signal network with positive or negative feedback loops and also regulated by compensatory mechanism. It is important to note that the apoptosis pathway is regulated by multiple cellular signaling. In cancer cells, the deregulations of multi-signaling and subsequent defective apoptosis often exist; therefore, targeting multiple signaling to induce apoptotic cell death by natural compound isoflavone could open a new avenue for cancer prevention and therapy as detailed in the following sections.

Isoflavones are mainly found in members of the Leguminosae family. Foods such as soy, lentil, bean, and chickpea are sources of isoflavones; however, soybean is the food that contains abundant amounts of isoflavones. Several epidemiological studies have shown that soy could have protective effects against prostate and other cancers [86,87]. The mostly investigated isoflavone is genistein. Experimental studies have revealed that isoflavone, particularly genistein, exerts its antioxidant effects on human cells. Isoflavone genistein protects cells against reactive oxygen species by scavenging free radicals and reducing the expression of stress-response related genes. Isoflavone genistein is also a well known tyrosine kinase inhibitor. We and other investigators have found that isoflavone genistein exerts its significant effects on the induction of apoptosis in several different types of cancers including lymphoma, leukemia, breast, prostate, lung, head and neck, and pancreatic cancers [88,89,90,91,92,93,94,95,96]. We also found that isoflavone genistein treatment increased the expression of Bax and p21, decreased the expression of Bcl-2 and Bcl_XL_, and activated PARP and caspases, leading to the induction of apoptotic cell death [89,90,91,92,95,96]. Moreover, isoflavone has been well known to induce apoptosis through the regulation of several important cellular signaling pathways as detailed in the following sections.

### 3.1. Isoflavone Induces Apoptosis through the Regulation of NF-κB Signaling

It is well known that NF-κB inhibits apoptosis. Therefore, we examined the status of NF-κB activity in isoflavone genistein treated breast, prostate, lung, and pancreatic cancer cells by electrophoresis mobility shift assay (EMSA) during our earlier studies [91,92,93,95,97]. We found that isoflavone genistein significantly inhibited the DNA-binding activity of NF-κB in various cancer cells. Furthermore, isoflavone genistein pre-treatment abrogated the activation of NF-κB stimulated by H_2_O_2_ or TNF-α. By immunochemistry and confocal microscopic analysis, we found that isoflavone, genistein inhibited the translocation of NF-κB to the nucleus, suggesting that isoflavone genistein may reduce the NF-κB binding to its target DNA and thereby inhibiting the transcription of its target genes such as Bcl-2, one of the important regulators of apoptosis. Recent studies have also shown that the induction of apoptosis by isoflavone, genistein and biochanin-A was mediated through both NF-κB dependent and independent pathways [98,99], suggesting the complexities of the biological activity of isoflavones.

To investigate the *in vivo *effects of isoflavone genistein on NF-κB signaling, we conducted *in vivo *study [100] where we found that when human volunteers received 50 mg of soy isoflavone supplements Novasoy™ (containing genistein, daidzein, and glycitein at a 1.3:1:0.3 ratio) twice daily for three weeks, TNF-α treatment *ex-vivo* failed to activate NF-κB activity in lymphocytes harvested from these volunteers, while lymphocytes from these volunteers collected prior to soy isoflavone intervention showed activation of NF-κB DNA binding activity upon TNF-α treatment *ex-vivo*. These results have demonstrated that soy isoflavone supplementation has a protective effect against TNF-α induced NF-κB activation in humans, suggesting that soy isoflavone could exert its cancer chemopreventive activity through the inhibition of NF-κB signaling and induction of apoptosis, which is required for elimination of damaged or pre-cancerous cells in order to maintain normal homeostasis (Figure 1). 

More importantly, we have conducted additional *in vitro *and *in vivo *studies to investigate whether isoflavone could enhance the anti-tumor and pro-apoptotic activities of chemotherapeutic agents. We pre-treated cancer cells or mice having cancers with isoflavone genistein and subsequently administrated conventional chemotherapeutic agents. We found that isoflavone genistein could increase apoptosis and enhance the anti-tumor activity of chemotherapeutic agents via the down-regulation of NF-κB signaling [93], suggesting the potential therapeutic effects of isoflavone in cancer treatment. Other investigators have also reported similar observations [101,102]. Considering the non-toxic nature and the potent activity on the induction of apoptosis, isoflavone-genistein could be useful in combination with conventional chemotherapeutic agents for achieving better treatment outcome of patients diagnosed with cancers. Further studies have shown that isoflavone could also enhance the effects of radiation therapy in pre-clinical models [103,104]. Interestingly, clinical study has shown that isoflavone could protect human prostate cancer patients from adverse side effects of traditional radiation therapy [105], suggesting the clinical significance of isoflavones. Thus, we believe that these results will help to initiate additional clinical trials using isoflavones for cancer prevention and/or treatment in the future.

### 3.2. Isoflavone Induces Apoptosis through the Regulation of Akt Signaling

Isoflavone genistein-induced apoptosis could also be regulated by Akt signaling, another signaling pathway involved in anti-apoptosis. To address this issue, we have conducted experiments to investigate the relationship between apoptosis, NF-κB, and Akt signaling [91]. By kinase activity assay and Western blot analysis, we found that isoflavone genistein did not alter the level of total Akt protein; however, the phosphorylated Akt protein at Ser473 and the Akt kinase activity were decreased after isoflavone genistein treatment. Isoflavone genistein pre-treatment also abrogated the activation of Akt by EGF. To further explore the inhibitory mechanism of isoflavone genistein on Akt and NF-κB pathways, we conducted gene transfection experiment, luciferase assay, and EMSA. By conducting these experiments, we have demonstrated that isoflavone genistein could exert its inhibitory effects on NF-κB pathway through Akt pathway. Furthermore, isoflavone genistein significantly induced apoptosis under the above experimental conditions. We also observed similar results in MDA-MB-231 breast cancer cells [97]. Therefore, the down-regulation of NF-κB and Akt signaling pathways by isoflavone genistein may be one of the molecular mechanisms by which isoflavone genistein induces apoptosis.

It is well known that activated Akt also inhibits apoptosis through the inhibition of GSK-3β and FOXO3a. We found that isoflavone genistein could inhibit the phosphorylation of Akt and FOXO3a, and up-regulated the expression of GSK-3β [94]. Isoflavone genistein also abrogated the phosphorylation of Akt and FOXO3a stimulated by IGF-1. Moreover, using four different apoptosis assays, we observed that isoflavone is an active agent to induce apoptosis, further abrogates the inhibition of apoptosis by IGF-1 treatment [94]. Other investigators recently reported similar findings showing that isoflavone genistein could induce apoptotic cell death through the regulation of Akt, NF-κB, and p21^WAF1^ [106,107]. All these results suggest that the regulation of Akt pathway by isoflavone genistein contributes to the induction of apoptosis (Figure 1).

### 3.3. Isoflavone Induces Apoptosis through the Regulation of MAPK Signaling

Isoflavone genistein has been found to inhibit the molecules in the MAPK pathway, leading to the induction of apoptosis. It has been reported that genistein could block the activation of p38 MAPK by TGF-β while p38 MAPK was necessary for TGF-β-mediated induction of MMP-2 and cell invasion in prostate cancer [108]. Therefore, genistein could inhibit cancer cell invasion and metastasis by blocking the activation of p38 MAPK. In another report, genistein treatment also triggered the inhibition of p38 MAPK activation in human hepatocellular carcinoma Hep3B cells [109]. Over-expression of p38 MAPK protected against apoptosis induced by combination treatment with genistein and TRAIL, suggesting that the p38 MAPK act as a key regulator of apoptosis in response to treatment with a combination of genistein and TRAIL [109]. In addition, synergistic anti-leukemia effect of genistein and chemotherapeutics in mouse xenograft model was found to be mediated through MAPK signaling [110]. Gene-expression profiling experiments revealed that MAPK signaling was one of the most affected biological pathways. MAPK kinase 4 was significantly down-regulated by genistein [110]. However, there are some controversial reports which showed that genistein potentiated apoptosis induced by arsenic trioxide in U937 human leukemia cells was regulated by ROS generation and p38 MAPK activation [111,112], suggesting that further in-depth investigations are needed to address these controversies.

### 3.4. Isoflavone Induces Apoptosis through the Regulation of Wnt Signaling

It has been known that Wnt signaling interacts with Akt signaling, promoting cancer cell proliferation and preventing cancer cells from apoptosis. We have reported that isoflavone up-regulated the expression of GSK-3β, enhanced GSK-3β binding to β-catenin, increased the phosphorylation of β-catenin, and induced apoptotic cell death, suggesting that isoflavone could inactivate Wnt signaling to induce apoptosis and inhibit prostate cancer cell growth [94]. Other investigators have also shown that isoflavone genistein decreased basal and Wnt-1-induced growth, and also inhibited the expression of Wnt-1 targets, c-Myc and Cyclin D1, [113], and that isoflavone down-regulated the expression of Wnt-5a [114] and Wnt-7a [115]. Animal experiments coupled with microarray gene expression analysis also demonstrated that isoflavone genistein down-regulated Wnt signaling in the tumor tissues of animals treated with isoflavone genistein [114]. Furthermore, the gene expression profiles showed decreased expression of Wnt target genes such as Cyclin D1, which is also a regulator of apoptosis, in mammary ductal epithelium of the animals treated with isoflavone genistein [114]. These findings suggest that isoflavone genistein could induce apoptosis and inhibit cancer cell growth through the regulation of Wnt signaling (Figure 1).

### 3.5. Isoflavone Induces Apoptosis through the Regulation of Notch Signaling

The effects of isoflavone genistein on apoptosis through Notch signaling have been documented in several reports. We have found that isoflavone genistein inhibited Notch signaling, leading to the down-regulation of NF-κB activity, the induction of apoptosis and the inhibition of cell proliferation in pancreatic cancer cells [72]. Other investigators have also reported that isoflavone, genistein could inhibit the expression of Notch-1 and Notch-2 [114,116], which is consistent with our findings. These results suggest that isoflavone genistein could inhibit cancer cell growth and induce apoptosis through the inhibition of Notch signaling pathways (Figure 1), which is intimately linked with NF-κB signaling pathway.

### 3.6. Isoflavone Induces Apoptosis through the Regulation of p53 Signaling

To investigate the effects of isoflavone genistein on p53 pathway, we measured cell growth inhibition, apoptosis, and gene expression related to apoptosis in genistein treated H460 lung cancer cells, which harbor wild type p53, and H322 lung cancer cells that possess a mutation in the *p53* gene [95,117]. Isoflavone genistein inhibited both H460 and H322 cell growth in a dose dependent manner and induced apoptosis in both cell lines, suggesting that the effects of isoflavone genistein is independent of p53 status. The expression of Bax and p21^WAF1^ was up-regulated in both H460 and H322 cells treated with isoflavone genistein, which clearly suggest p53-independent pathway for the activation of apoptosis. Importantly, increased wild-type p53 protein was found in genistein-treated H460 cells, while no change in p53 expression was observed in H322 cells treated with isoflavone genistein. These results suggest that isoflavone genistein induces apoptosis in lung cancer cells through both p53-dependent and independent pathways. Isoflavone treatment also stabilized p53, leading to the inactivation of Bcl-2 and the induction of apoptosis in MCF-7 breast cancer cells [118]. Isoflavone genistein could also protect p53 wild type cells from toxicity and selectively increase apoptosis in p53-deficient cells [119]. Moreover, experimental studies have shown that isoflavone genistein treatment combined with sorafenib or perifosine could cause increased apoptosis and growth arrest through up-regulation of p53 and p21^WAF1^ [120,121]. These findings suggest that isoflavone could induce apoptosis by up-regulation of p53 signaling (Figure 1); however, the role of p53 in the context of the biological activity of isofalvone has many layers of complexity.

### 3.7. Isoflavone Induces Apoptosis through the Regulation of AR Signaling

AR signaling is also a target of isoflavone genistein during the induction of apoptosis (Figure 1). We have reported that isoflavone genistein transcriptionally down-regulated AR, inhibited nuclear AR binding to androgen responsive element, and thereby decreased the transcription and protein expression of PSA in androgen-sensitive LNCaP cells, leading to the induction of apoptosis and the inhibition of cancer cell growth [122]. Similar findings has also been reported showing that isoflavone significantly down-regulated AR signaling with decreased AR protein levels and a consequent reduction in transcriptional activity and expression of PSA [123]. By mechanistic studies, we found that isoflavone-induced inhibition of cell proliferation and induction of apoptosis are partly mediated through the regulation of the Akt/FOXO3a/GSK-3β/AR signaling network [94]. The *in vivo* animal study also showed that dietary genistein down-regulated the expression of AR in the rat prostate at concentrations comparable to those found in humans on a soy diet [124]. Based on these results, we believe that the down-regulation of AR expression by isoflavone to induce apoptotic cell death could be an important strategy for the prevention and/or treatment of prostate cancer, especially castrate resistant prostate cancer for which there is no curative treatment.

### 3.8. Isoflavone Regulates miRNAs, Leading to Increased Drug Sensitivity and Cancer Cell Death

Emerging evidence has shown that natural agents including isoflavone could regulate the expression of specific miRNAs [125,126], which may increase sensitivity of cancer cells to conventional chemotherapeutics and, thereby, lead to increased cancer cell death induced by chemotherapeutics. Considering the non-toxic characteristics of isoflavone, targeting miRNAs by isoflavone combined with conventional chemotherapeutics could be a novel and safer approach for achieving better treatment outcome of patients diagnosed with cancers. It is well known that the aggressiveness of pancreatic cancer is in part due to its drug resistance characteristics, which are associated with pancreatic cancer stem-like cells and the acquisition of EMT phenotype. Therefore, we investigated the regulatory effects of isoflavone on miRNAs in pancreatic cancer cells that are gemcitabine-resistant and have the typical EMT phenotype that lost the expression of miR-200. We found that re-expression of miR-200 by pre-miR-200 transfection or treatment of gemcitabine-resistant cells with isoflavone could cause up-regulation in the expression of miR-200, resulting in the down-regulation of ZEB1, slug, and vimentin, which were consistent with morphologic reversal of EMT phenotype leading to epithelial morphology [125]. Importantly, we found that miR-200 re-expression or isoflavone treatment increased sensitivity of gemcitabine-resistant pancreatic cells to gemcitabine, leading to increased pancreatic cancer cell death induced by gemcitabine [125]. These results suggest that miR-200 re-expression or isoflavone treatment could partially increase the sensitivity of gemcitabine-resistant cells to gemcitabine possibly through miR-200 mediated reversal of EMT status. A recent study has also shown that isoflavone, genistein could down-regulate miR-221/222, leading to the up-regulation of ARHI, one of the tumor suppressors, causing induction of apoptosis [127]. Therefore, conventional chemotherapeutics combined with isoflavone could be a novel strategy for achieving better treatment outcome of patients diagnosed with pancreatic and other cancers for which future in-depth mechanistic and clinical studies are warranted.

## 4. Summary and Perspectives

The data from *in vivo* human and animal studies and *in vitro* experiments clearly suggest that isoflavone exerts its inhibitory effects on carcinogenesis and cancer progression by induction of apoptosis and inhibition of cell proliferation. The induction of apoptosis by isoflavone has been believed to be mediated through the regulation of multiple cell signaling pathways including NF-κB, Akt, MAPK, Wnt, Notch, p53, and AR signaling pathways. Because of the complex communication between cell signaling networks, cancer cells always show alterations in multiple cellular signaling pathways. Therefore, regulation of multiple cell signaling pathways for controlling the behavior of cancer cells such as apoptosis requires agents that could target multiple pathways. It has now been well recognized that isoflavone could target multiple pathways to induce apoptotic cell death; therefore, we believe that isoflavone could be useful either alone or in combination with conventional therapeutics (chemotherapy and radiotherapy) for the prevention of tumor progression and/or treatment of most human malignancies. A number of phase I and II clinical trials have been conducted to investigate the toxicity and effects of isoflavones in healthy men and in patients with prostate cancer [128]. The results demonstrated that isoflavones could be administrated safely and that isoflavones were able to alter markers of cancer cell proliferation such as serum PSA and free testosterone in a larger number of subjects. Currently, several clinical trials are being conducted using isoflavone genistein, formulated genistein, or isoflavone analogue phenoxodiol in combination with IL-2, gemcitabine, docetaxel or cisplatin in the treatment of melanoma, kidney, ovarian and pancreatic cancers [128], suggesting the potential benefits of isoflavone in cancer in the clinical setting. However, further in-depth mechanistic studies *in vitro*, relevant animal model studies *in vivo* and novel clinical trials are needed in order to better understand the molecular mechanisms of isoflavone action, and to further realize the value of isoflavone and/or its synthetic analogs for the prevention of tumor progression and/or treatment of human malignancies.

## References

[1] Meier P., Finch A., Evan G. (2000). Apoptosis in development.. Nature.

[2] Zhou Z., Sun X., Kang Y.J. (2001). Ethanol-induced apoptosis in mouse liver: Fas- and cytochrome c-mediated caspase-3 activation pathway.. Am. J. Pathol..

[3] Shi Y. (2004). Caspase activation: Revisiting the induced proximity model.. Cell.

[4] Danial N.N., Korsmeyer S.J. (2004). Cell death: Critical control points.. Cell.

[5] Boulares A.H., Yakovlev A.G., Ivanova V., Stoica B.A., Wang G., Iyer S., Smulson M. (1999). Role of poly(ADP-ribose) polymerase (PARP) cleavage in apoptosis. Caspase 3-resistant PARP mutant increases rates of apoptosis in transfected cells.. J. Biol. Chem..

[6] Simbulan-Rosenthal C.M., Rosenthal D.S., Iyer S., Boulares H., Smulson M.E. (1999). Involvement of PARP and poly(ADP-ribosyl)ation in the early stages of apoptosis and DNA replication.. Mol. Cell. Biochem..

[7] Ruvolo P.P., Deng X., May W.S. (2001). Phosphorylation of Bcl2 and regulation of apoptosis.. Leukemia.

[8] Kitada S., Pedersen I.M., Schimmer A.D., Reed J.C. (2002). Dysregulation of apoptosis genes in hematopoietic malignancies.. Oncogene.

[9] Vousden K.H., Lu X. (2002). Live or let die: The cell’s response to p53.. Nat. Rev. Cancer.

[10] Yakovlev A.G., Wang G., Stoica B.A., Boulares H.A., Spoonde A.Y., Yoshihara K., Smulson M.E. (2000). A role of the Ca^2+^/Mg^2+^-dependent endonuclease in apoptosis and its inhibition by Poly(ADP-ribose) polymerase.. J. Biol. Chem..

[11] Guo Y., Srinivasula S.M., Druilhe A., Fernandes-Alnemri T., Alnemri E.S. (2002). Caspase-2 induces apoptosis by releasing proapoptotic proteins from mitochondria.. J. Biol. Chem..

[12] Evan G.I., Vousden K.H. (2001). Proliferation, cell cycle and apoptosis in cancer. Nature.

[13] Kasibhatla S., Tseng B. (2003). Why target apoptosis in cancer treatment?. Mol. Cancer Ther..

[14] Shaw A.S., Filbert E.L. (2009). Scaffold proteins and immune-cell signalling.. Nat. Rev. Immunol..

[15] Martin G.S. (2003). Cell signaling and cancer.. Cancer Cell.

[16] Karin M., Cao Y., Greten F.R., Li Z.W. (2002). NF-kappaB in cancer: From innocent bystander to major culprit.. Nat. Rev. Cancer.

[17] Klaus A., Birchmeier W. (2008). Wnt signalling and its impact on development and cancer.. Nat. Rev. Cancer.

[18] Sebolt-Leopold J.S., Herrera R. (2004). Targeting the mitogen-activated protein kinase cascade to treat cancer.. Nat. Rev. Cancer.

[19] Stiewe T. (2007). The p53 family in differentiation and tumorigenesis.. Nat. Rev. Cancer.

[20] Igney F.H., Krammer P.H. (2002). Death and anti-death: tumour resistance to apoptosis.. Nat. Rev. Cancer.

[21] Lee M.M., Gomez S.L., Chang J.S., Wey M., Wang R.T., Hsing A.W. (2003). Soy and isoflavone consumption in relation to prostate cancer risk in China.. Cancer Epidemiol. Biomark. Prev..

[22] Mukhtar H., Ahmad N. (1999). Green tea in chemoprevention of cancer.. Toxicol. Sci..

[23] Smith-Warner S.A., Spiegelman D., Yaun S.S., Albanes D., Beeson W.L., van den Brandt P.A., Feskanich D., Folsom A.R., Fraser G.E., Freudenheim J.L. (2003). Fruits, vegetables and lung cancer: a pooled analysis of cohort studies.. Int. J. Cancer.

[24] Surh Y.J. (2003). Cancer chemoprevention with dietary phytochemicals.. Nat. Rev. Cancer.

[25] Khan N., Afaq F., Mukhtar H. (2007). Apoptosis by dietary factors: The suicide solution for delaying cancer growth.. Carcinogenesis.

[26] Lamartiniere C.A., Cotroneo M.S., Fritz W.A., Wang J., Mentor-Marcel R., Elgavish A. (2002). Genistein chemoprevention: timing and mechanisms of action in murine mammary and prostate.. J. Nutr..

[27] Li Y., Li X., Sarkar F.H. (2003). Gene expression profiles of I3C- and DIM-treated PC3 human prostate cancer cells determined by cDNA microarray analysis.. J. Nutr..

[28] Gupta S., Hussain T., Mukhtar H. (2003). Molecular pathway for (−)-epigallocatechin-3-gallate-induced cell cycle arrest and apoptosis of human prostate carcinoma cells.. Arch. Biochem. Biophys..

[29] Hastak K., Gupta S., Ahmad N., Agarwal M.K., Agarwal M.L., Mukhtar H. (2003). Role of p53 and NF-kappaB in epigallocatechin-3-gallate-induced apoptosis of LNCaP cells.. Oncogene.

[30] Dixon R.A., Ferreira D. (2002). Genistein.. Phytochemistry.

[31] Li Y., Sarkar F.H. (2002). Down-regulation of invasion and angiogenesis-related genes identified by cDNA microarray analysis of PC3 prostate cancer cells treated with genistein.. Cancer Lett..

[32] Storz P., Toker A. (2003). NF-kappaB signaling-an alternate pathway for oxidative stress responses.. Cell Cycle.

[33] Lin A., Karin M. (2003). NF-kappaB in cancer: a marked target.. Semin. Cancer Biol..

[34] Haefner B. (2002). NF-kappaB: Arresting a major culprit in cancer.. Drug Discov. Today.

[35] Orlowski R.Z., Baldwin A.S. (2002). NF-kappaB as a therapeutic target in cancer.. Trends Mol. Med..

[36] Clarkson R.W., Heeley J.L., Chapman R., Aillet F., Hay R.T., Wyllie A., Watson C.J. (2000). NF-kappaB inhibits apoptosis in murine mammary epithelia.. J. Biol. Chem..

[37] Arlt A., Vorndamm J., Breitenbroich M., Folsch U.R., Kalthoff H., Schmidt W.E., Schafer H. (2001). Inhibition of NF-kappaB sensitizes human pancreatic carcinoma cells to apoptosis induced by etoposide (VP16) or doxorubicin.. Oncogene.

[38] Mitsiades C.S., Mitsiades N., Poulaki V., Schlossman R., Akiyama M., Chauhan D., Hideshima T., Treon S.P., Munshi N.C., Richardson P.G., Anderson K.C. (2002). Activation of NF-kappaB and upregulation of intracellular anti-apoptotic proteins via the IGF-1/Akt signaling in human multiple myeloma cells: Therapeutic implications.. Oncogene.

[39] Rogers R., Ouellet G., Brown C., Moyer B., Rasoulpour T., Hixon M. (2008). Cross-talk between the Akt and NF-kappaB signaling pathways inhibits MEHP-induced germ cell apoptosis.. Toxicol. Sci..

[40] Clarke R.B. (2003). p27KIP1 phosphorylation by PKB/Akt leads to poor breast cancer prognosis.. Breast Cancer Res..

[41] Chang F., Lee J.T., Navolanic P.M., Steelman L.S., Shelton J.G., Blalock W.L., Franklin R.A., McCubrey J.A. (2003). Involvement of PI3K/Akt pathway in cell cycle progression, apoptosis, and neoplastic transformation: A target for cancer chemotherap. Leukemia.

[42] Alessi D.R., Andjelkovic M., Caudwell B., Cron P., Morrice N., Cohen P., Hemmings B.A. (1996). Mechanism of activation of protein kinase B by insulin and IGF-1.. EMBO J..

[43] Brunet A., Bonni A., Zigmond M.J., Lin M.Z., Juo P., Hu L.S., Anderson M.J., Arden K.C., Blenis J., Greenberg M.E. (1999). Akt promotes cell survival by phosphorylating and inhibiting a Forkhead transcription factor.. Cell.

[44] Cardone M.H., Roy N., Stennicke H.R., Salvesen G.S., Franke T.F., Stanbridge E., Frisch S., Reed J.C. (1998). Regulation of cell death protease caspase-9 by phosphorylation.. Science.

[45] Rommel C., Clarke B.A., Zimmermann S., Nunez L., Rossman R., Reid K., Moelling K., Yancopoulos G.D., Glass D.J. (1999). Differentiation stage-specific inhibition of the Raf-MEK-ERK pathway by Akt.. Science.

[46] Ozes O.N., Mayo L.D., Gustin J.A., Pfeffer S.R., Pfeffer L.M., Donner D.B. (1999). NF-kappaB activation by tumour necrosis factor requires the Akt serine-threonine kinase.. Nature.

[47] Romashkova J.A., Makarov S.S. (1999). NF-kappaB is a target of AKT in anti-apoptotic PDGF signalling.. Nature.

[48] Hill M.M., Hemmings B.A. (2002). Inhibition of protein kinase B/Akt. implications for cancer therapy.. Pharmacol. Ther..

[49] Sebolt-Leopold J.S. (2000). Development of anticancer drugs targeting the MAP kinase pathway.. Oncogene.

[50] Seger R., Krebs E.G. (1995). The MAPK signaling cascade.. FASEB J..

[51] Fang J.Y., Richardson B.C. (2005). The MAPK signalling pathways and colorectal cancer.. Lancet Oncol..

[52] Lunghi P., Giuliani N., Mazzera L., Lombardi G., Ricca M., Corradi A., Cantoni A.M., Salvatore L., Riccioni R., Costanzo A. (2008). Targeting MEK/MAPK signal transduction module potentiates ATO-induced apoptosis in multiple myeloma cells through multiple signaling pathways.. Blood.

[53] Zhang Y., Ting A.T., Marcu K.B., Bliska J.B. (2005). Inhibition of MAPK and NF-kappa B pathways is necessary for rapid apoptosis in macrophages infected with Yersinia.. J. Immunol..

[54] van Laethem A., van Kelst S., Lippens S., Declercq W., Vandenabeele P., Janssens S., Vandenheede J.R., Garmyn M., Agostinis P. (2004). Activation of p38 MAPK is required for Bax translocation to mitochondria, cytochrome c release and apoptosis induced by UVB irradiation in human keratinocytes. FASEB J..

[55] Tanaka Y., Gavrielides M.V., Mitsuuchi Y., Fujii T., Kazanietz M.G. (2003). Protein kinase C promotes apoptosis in LNCaP prostate cancer cells through activation of p38 MAPK and inhibition of the Akt survival pathway.. J. Biol. Chem..

[56] Angers S., Moon R.T. (2009). Proximal events in Wnt signal transduction.. Nat. Rev. Mol. Cell Biol..

[57] Behrens J. (2000). Control of beta-catenin signaling in tumor development.. Ann. N. Y. Acad. Sci..

[58] Peifer M., Polakis P. (2000). Wnt signaling in oncogenesis and embryogenesis-a look outside the nucleus.. Science.

[59] Reya T., Clevers H. (2005). Wnt signalling in stem cells and cancer.. Nature.

[60] Dehner M., Hadjihannas M., Weiske J., Huber O., Behrens J. (2008). Wnt signaling inhibits Forkhead box O3a-induced transcription and apoptosis through up-regulation of serum- and glucocorticoid-inducible kinase 1.. J. Biol. Chem..

[61] Almeida M., Han L., Bellido T., Manolagas S.C., Kousteni S. (2005). Wnt proteins prevent apoptosis of both uncommitted osteoblast progenitors and differentiated osteoblasts by beta-catenin-dependent and -independent signaling cascades involving Src/ERK and phosphatidylinositol 3-kinase/AKT.. J. Biol. Chem..

[62] He B., Reguart N., You L., Mazieres J., Xu Z., Lee A.Y., Mikami I., McCormick F., Jablons D.M. (2005). Blockade of Wnt-1 signaling induces apoptosis in human colorectal cancer cells containing downstream mutations.. Oncogene.

[63] You L., He B., Xu Z., Uematsu K., Mazieres J., Fujii N., Mikami I., Reguart N., McIntosh J.K., Kashani-Sabet M., McCormick F., Jablons D.M. (2004). An anti-Wnt-2 monoclonal antibody induces apoptosis in malignant melanoma cells and inhibits tumor growth.. Cancer Res..

[64] Dihlmann S., von Knebel D.M. (2005). Wnt/beta-catenin-pathway as a molecular target for future anti-cancer therapeutics.. Int. J. Cancer.

[65] Barker N., Clevers H. (2006). Mining the Wnt pathway for cancer therapeutics.. Nat. Rev. Drug Discov..

[66] Rizzo P., Osipo C., Foreman K., Golde T., Osborne B., Miele L. (2008). Rational targeting of Notch signaling in cancer.. Oncogene.

[67] Zardawi S.J., O’Toole S.A., Sutherland R.L., Musgrove E.A. (2009). Dysregulation of hedgehog, Wnt and notch signalling pathways in breast cancer. Histol. Histopathol..

[68] Wang Z., Li Y., Banerjee S., Sarkar F.H. (2009). Emerging role of Notch in stem cells and cancer.. Cancer Lett..

[69] Wang Z., Banerjee S., Li Y., Rahman K.M., Zhang Y., Sarkar F.H. (2006). Down-regulation of notch-1 inhibits invasion by inactivation of nuclear factor-kappaB, vascular endothelial growth factor, and matrix metalloproteinase-9 in pancreatic cancer cell. Cancer Res..

[70] Meurette O., Stylianou S., Rock R., Collu G.M., Gilmore A.P., Brennan K. (2009). Notch activation induces Akt signaling via an autocrine loop to prevent apoptosis in breast epithelial cells.. Cancer Res..

[71] Liu W.H., Hsiao H.W., Tsou W.I., Lai M.Z. (2007). Notch inhibits apoptosis by direct interference with XIAP ubiquitination and degradation.. EMBO J..

[72] Wang Z., Zhang Y., Li Y., Banerjee S., Liao J., Sarkar F.H. (2006). Down-regulation of Notch-1 contributes to cell growth inhibition and apoptosis in pancreatic cancer cells.. Mol. Cancer Ther..

[73] Wang Z., Li Y., Banerjee S., Kong D., Ahmad A., Nogueira V., Hay N., Sarkar F.H. (2010). Down-regulation of Notch-1 and Jagged-1 inhibits prostate cancer cell growth, migration and invasion, and induces apoptosis via inactivation of Akt, mTOR, and NF-kappaB signaling pathways. J. Cell. Biochem..

[74] Nefedova Y., Sullivan D.M., Bolick S.C., Dalton W.S., Gabrilovich D.I. (2008). Inhibition of Notch signaling induces apoptosis of myeloma cells and enhances sensitivity to chemotherapy.. Blood.

[75] Zweidler-McKay P.A., He Y., Xu L., Rodriguez C.G., Karnell F.G., Carpenter A.C., Aster J.C., Allman D., Pear W.S. (2005). Notch signaling is a potent inducer of growth arrest and apoptosis in a wide range of B-cell malignancies.. Blood.

[76] Chadwick N., Fennessy C., Nostro M.C., Baron M., Brady G., Buckle A.M. (2008). Notch induces cell cycle arrest and apoptosis in human erythroleukaemic TF-1 cells.. Blood Cells Mol. Dis..

[77] Manic S., Gatti L., Carenini N., Fumagalli G., Zunino F., Perego P. (2003). Mechanisms controlling sensitivity to platinum complexes: Role of p53 and DNA mismatch repair.. Curr. Cancer Drug Targets.

[78] Luke M.C., Coffey D.S. (1994). Human androgen receptor binding to the androgen response element of prostate specific antigen.. J. Androl..

[79] Montgomery J.S., Price D.K., Figg W.D. (2001). The androgen receptor gene and its influence on the development and progression of prostate cancer.. J. Pathol..

[80] Kupelian P., Katcher J., Levin H., Zippe C., Klein E. (1996). Correlation of clinical and pathologic factors with rising prostate-specific antigen profiles after radical prostatectomy alone for clinically localized prostate cancer.. Urology.

[81] Sato N., Gleave M.E., Bruchovsky N., Rennie P.S., Goldenberg S.L., Lange P.H., Sullivan L.D. (1996). Intermittent androgen suppression delays progression to androgen-independent regulation of prostate-specific antigen gene in the LNCaP prostate tumour model.. J. Steroid Biochem. Mol. Biol..

[82] Heinlein C.A., Chang C. (2004). Androgen receptor in prostate cancer.. Endocr. Rev..

[83] Lin Y., Kokontis J., Tang F., Godfrey B., Liao S., Lin A., Chen Y., Xiang J. (2006). Androgen and its receptor promote Bax-mediated apoptosis.. Mol. Cell. Biol..

[84] Chiu F.L., Lin J.K. (2008). Downregulation of androgen receptor expression by luteolin causes inhibition of cell proliferation and induction of apoptosis in human prostate cancer cells and xenografts.. Prostate.

[85] Chen D., Cui Q.C., Yang H., Barrea R.A., Sarkar F.H., Sheng S., Yan B., Reddy G.P., Dou Q.P. (2007). Clioquinol, a therapeutic agent for Alzheimer’s disease, has proteasome-inhibitory, androgen receptor-suppressing, apoptosis-inducing, and antitumor activities in human prostate cancer cells and xenografts.. Cancer Res..

[86] Hebert J.R., Hurley T.G., Olendzki B.C., Teas J., Ma Y., Hampl J.S. (1998). Nutritional and socioeconomic factors in relation to prostate cancer mortality: a cross-national study.. J. Natl. Cancer Inst..

[87] Jacobsen B.K., Knutsen S.F., Fraser G.E. (1998). Does high soy milk intake reduce prostate cancer incidence? The adventist health study (United States).. Cancer Causes Control.

[88] Wang Z., Li Y., Ahmad A., Banerjee S., Azmi A.S., Kong D., Wojewoda C., Miele L., Sarkar F.H. (2010). Down-regulation of Notch-1 is associated with Akt and FoxM1 in inducing cell growth inhibition and apoptosis in prostate cancer cells.. J. Cell. Biochem..

[89] Alhasan S.A., Aranha O., Sarkar F.H. (2001). Genistein elicits pleiotropic molecular effects on head and neck cancer cells.. Clin. Cancer Res..

[90] Li Y., Upadhyay S., Bhuiyan M., Sarkar F.H. (1999). Induction of apoptosis in breast cancer cells MDA-MB-231 by genistein.. Oncogene.

[91] Li Y., Sarkar F.H. (2002). Inhibition of nuclear factor kappaB activation in PC3 cells by genistein is mediated via Akt signaling pathway.. Clin. Cancer Res..

[92] Li Y., Ellis K.L., Ali S., El-Rayes B.F., Nedeljkovic-Kurepa A., Kucuk O., Philip P.A., Sarkar F.H. (2004). Apoptosis-inducing effect of chemotherapeutic agents is potentiated by soy isoflavone genistein, a natural inhibitor of NF-kappaB in BxPC-3 pancreatic cancer cell line. Pancreas.

[93] Li Y., Ahmed F., Ali S., Philip P.A., Kucuk O., Sarkar F.H. (2005). Inactivation of nuclear factor kappaB by soy isoflavone genistein contributes to increased apoptosis induced by chemotherapeutic agents in human cancer cells.. Cancer Res..

[94] Li Y., Wang Z., Kong D., Li R., Sarkar S.H., Sarkar F.H. (2008). Regulation of Akt/FOXO3a/GSK-3beta/AR signaling network by isoflavone in prostate cancer cells.. J. Biol. Chem..

[95] Lian F., Bhuiyan M., Li Y.W., Wall N., Kraut M., Sarkar F.H. (1998). Genistein-induced G2-M arrest, p21WAF1 upregulation, and apoptosis in a non-small-cell lung cancer cell line.. Nutr. Cancer.

[96] Baxa D.M., Yoshimura F.K. (2003). Genistein reduces NF-kappa B in T lymphoma cells via a caspase-mediated cleavage of I kappa B alpha.. Biochem. Pharmacol..

[97] Gong L., Li Y., Nedeljkovic-Kurepa A., Sarkar F.H. (2003). Inactivation of NF-kappaB by genistein is mediated via Akt signaling pathway in breast cancer cells.. Oncogene.

[98] Lee Y.K., Park O.J. (2011). Soybean isoflavone genistein regulates apoptosis through NF-kappaB dependent and independent pathways.. Exp. Toxicol. Pathol..

[99] Kole L., Giri B., Manna S.K., Pal B., Ghosh S. (2011). Biochanin-A, an isoflavon, showed anti-proliferative and anti-inflammatory activities through the inhibition of iNOS expression, p38-MAPK and ATF-2 phosphorylation and blocking NFkappaB nuclear translocation.. Eur. J. Pharmacol..

[100] Davis J.N., Kucuk O., Djuric Z., Sarkar F.H. (2001). Soy isoflavone supplementation in healthy men prevents NF-kappa B activation by TNF-alpha in blood lymphocytes.. Free Radic. Biol. Med..

[101] Satoh H., Nishikawa K., Suzuki K., Asano R., Virgona N., Ichikawa T., Hagiwara K., Yano T. (2003). Genistein, a soy isoflavone, enhances necrotic-like cell death in a breast cancer cell treated with a chemotherapeutic agent.. Res. Commun. Mol. Pathol. Pharmacol..

[102] Khoshyomn S., Manske G.C., Lew S.M., Wald S.L., Penar P.L. (2000). Synergistic action of genistein and cisplatin on growth inhibition and cytotoxicity of human medulloblastoma cells.. Pediatr. Neurosurg..

[103] Singh-Gupta V., Zhang H., Yunker C.K., Ahmad Z., Zwier D., Sarkar F.H., Hillman G.G. (2010). Daidzein effect on hormone refractory prostate cancer in vitro and in vivo compared to genistein and soy extract: potentiation of radiotherapy.. Pharm. Res..

[104] Singh-Gupta V., Zhang H., Banerjee S., Kong D., Raffoul J.J., Sarkar F.H., Hillman G.G. (2009). Radiation-induced HIF-1alpha cell survival pathway is inhibited by soy isoflavones in prostate cancer cells.. Int. J. Cancer.

[105] Ahmad I.U., Forman J.D., Sarkar F.H., Hillman G.G., Heath E., Vaishampayan U., Cher M.L., Andic F., Rossi P.J., Kucuk O. (2010). Soy isoflavones in conjunction with radiation therapy in patients with prostate cancer.. Nutr. Cancer.

[106] Ma Y., Wang J., Liu L., Zhu H., Chen X., Pan S., Sun X., Jiang H. (2011). Genistein potentiates the effect of arsenic trioxide against human hepatocellular carcinoma: Role of Akt and nuclear factor-kappaB.. Cancer Lett..

[107] Privat M., Aubel C., Arnould S., Communal Y., Ferrara M., Bignon Y.J. (2010). AKT and p21 WAF1/CIP1 as potential genistein targets in BRCA1-mutant human breast cancer cell lines.. Anticancer Res..

[108] Huang X., Chen S., Xu L., Liu Y., Deb D.K., Platanias L.C., Bergan R.C. (2005). Genistein inhibits p38 map kinase activation, matrix metalloproteinase type 2, and cell invasion in human prostate epithelial cell. Cancer Res..

[109] Jin C.Y., Park C., Kim G.Y., Lee S.J., Kim W.J., Choi Y.H. (2009). Genistein enhances TRAIL-induced apoptosis through inhibition of p38 MAPK signaling in human hepatocellular carcinoma Hep3B cells.. Chem. Biol. Interact..

[110] Shen J., Tai Y.C., Zhou J., Stephen Wong C.H., Cheang P.T., Fred Wong W.S., Xie Z., Khan M., Han J.H., Chen C.S. (2007). Synergistic antileukemia effect of genistein and chemotherapy in mouse xenograft model and potential mechanism through MAPK signaling.. Exp. Hematol..

[111] Sánchez Y., Calle C., de Blas E., Aller P. (2009). Modulation of arsenic trioxide-induced apoptosis by genistein and functionally related agents in U937 human leukaemia cells. Regulation by ROS and mitogen-activated protein kinases.. Chem. Biol. Interact..

[112] Sánchez Y., Amran D., Fernandez C., de Blas E., Aller P. (2008). Genistein selectively potentiates arsenic trioxide-induced apoptosis in human leukemia cells via reactive oxygen species generation and activation of reactive oxygen species-inducible protein kinases (p38-MAPK, AMPK). Int. J. Cancer.

[113] Su Y., Simmen R.C. (2009). Soy isoflavone genistein up-regulates epithelial adhesion molecule E-cadherin expression and attenuates beta-catenin signaling in mammary epithelial cells.. Carcinogenesis.

[114] Su Y., Simmen F.A., Xiao R., Simmen R.C. (2007). Expression profiling of rat mammary epithelial cells reveals candidate signaling pathways in dietary protection from mammary tumors.. Physiol. Genomics.

[115] Wagner J., Lehmann L. (2006). Estrogens modulate the gene expression of Wnt-7a in cultured endometrial adenocarcinoma cells.. Mol. Nutr. Food Res..

[116] Janardhanan R., Banik N.L., Ray S.K. (2009). N-Myc down regulation induced differentiation, early cell cycle exit, and apoptosis in human malignant neuroblastoma cells having wild type or mutant p5. Biochem. Pharmacol..

[117] Lian F., Li Y., Bhuiyan M., Sarkar F.H. (1999). p53-independent apoptosis induced by genistein in lung cancer cells.. Nutr. Cancer.

[118] Constantinou A.I., Kamath N., Murley J.S. (1998). Genistein inactivates Bcl-2, delays the G2/M phase of the cell cycle, and induces apoptosis of human breast adenocarcinoma MCF-7 cell. Eur. J. Cancer.

[119] Tokalov S.V., Abramyuk A.M., Abolmaali N.D. (2010). Protection of p53 wild type cells from taxol by genistein in the combined treatment of lung cancer.. Nutr. Cancer.

[120] Roy C.S., Karmakar S., Banik N.L., Ray S.K. (2010). Synergistic efficacy of sorafenib and genistein in growth inhibition by down regulating angiogenic and survival factors and increasing apoptosis through upregulation of p53 and p21 in malignant neuroblastoma cells having N-Myc amplification or non-amplification.. Invest. New Drugs.

[121] Vinall R.L., Hwa K., Ghosh P., Pan C.X., Lara P.N., de Vere White R.W. (2007). Combination treatment of prostate cancer cell lines with bioactive soy isoflavones and perifosine causes increased growth arrest and/or apoptosis. Clin. Cancer Res..

[122] Davis J.N., Kucuk O., Sarkar F.H. (2002). Expression of prostate-specific antigen is transcriptionally regulated by genistein in prostate cancer cells.. Mol. Carcinog..

[123] Tepper C.G., Vinall R.L., Wee C.B., Xue L., Shi X.B., Burich R., Mack P.C., de Vere White R.W. (2007). GCP-mediated growth inhibition and apoptosis of prostate cancer cells via androgen receptor-dependent and -independent mechanisms.. Prostate.

[124] Fritz W.A., Wang J., Eltoum I.E., Lamartiniere C.A. (2002). Dietary genistein down-regulates androgen and estrogen receptor expression in the rat prostate.. Mol. Cell. Endocrinol..

[125] Li Y., VandenBoom T.G., Kong D., Wang Z., Ali S., Philip P.A., Sarkar F.H. (2009). Up-regulation of miR-200 and let-7 by natural agents leads to the reversal of epithelial-to-mesenchymal transition in gemcitabine-resistant pancreatic cancer cells.. Cancer Res..

[126] Tsang W.P., Kwok T.T. (2010). Epigallocatechin gallate up-regulation of miR-16 and induction of apoptosis in human cancer cells.. J. Nutr. Biochem..

[127] Chen Y., Zaman M.S., Deng G., Majid S., Saini S., Liu J., Tanaka Y., Dahiya R. (2011). MicroRNAs 221/222 and genistein-mediated regulation of ARHI tumor suppressor gene in prostate cancer.. Cancer Prev. Res. (Phila.).

[128] ClinicalTrial.org. http://www.clinicaltrials.gov/ct2/results?term=Isoflavone+AND+cancer.

